# Trajectories of Objectively Measured Physical Activity among Secondary Students in Canada in the Context of a Province-Wide Physical Education Policy: A Longitudinal Analysis

**DOI:** 10.1155/2014/958645

**Published:** 2014-01-20

**Authors:** Erin Hobin, Jannice So, Laura Rosella, Melisa Comte, Steve Manske, Jonathan McGavock

**Affiliations:** ^1^Public Health Ontario, 480 University Avenue, Suite 300, Toronto, ON, Canada M5G 1V2; ^2^Manitoba Institute of Child Health, 513-715 McDermot Avenue, Winnipeg, MB, Canada R3E 3P4; ^3^Propel Centre for Population Health Impact, University of Waterloo, 200 University Avenue West, Waterloo, ON, Canada N2L 3G1

## Abstract

Lower levels of physical activity are associated with childhood obesity. School physical education (PE) policies have been identified as critical to improve child and adolescent physical activity levels but there has been little evaluation of such policies. In the province of Manitoba, Canada, the government implemented a mandatory PE policy in secondary schools designed to increase the daily physical activity levels of adolescents. The objective of this study was to examine the longitudinal changes in and the factors associated with the physical activity trajectories of adolescents in Manitoba during their tenure as secondary school students in the context of this school PE policy. The results found, despite the PE policy, a grade-related decline in the physical activity trajectories of adolescents; however, the decline in physical activity was attenuated among adolescents with low and moderate baseline physical activity compared to adolescents with high baseline physical activity and among adolescents who attended schools in neighbourhoods of low compared to high socioeconomic status. There are several possible explanations for these findings, including the influence of the PE policy on the PA patterns of adolescent subpopulations that tend to be at higher risk for inactivity in both childhood and adult life.

## 1. Introduction

The prevalence of overweight and obesity has risen dramatically among Canadian youth. Since 1981, childhood obesity rates have almost tripled, with approximately 31.5% of Canadians aged 5–17 years currently being overweight or obese [1]. Childhood obesity is an important predictor of adult obesity and significantly increases the risk of chronic disease, including type 2 diabetes and cardiovascular disease [2, 3].

Increasing physical activity (PA) to levels recommended by experts can contribute to lowering the risk for overweight and obesity among children and adolescents [4]. Despite the protective link between PA and obesity, recent surveillance studies suggest that up to 93% of Canadians aged 6–19 years do not achieve the recommended dose of PA required for adequate growth and health [5]. Furthermore, the rates of inactive youth increase rapidly in the years following puberty and reach a pinnacle in adolescence where PA rates can decline by as much as 85% by the age of 15 years [6, 7]. The rates of obesity are also highest during this period of development (15–18 years) [8]. As physical inactivity in adolescence predicts PA patterns in adulthood and physical inactivity is associated with a cost of $4 billion to the Canadian economy, public health experts are calling for strategies to increase PA rates among adolescents [9, 10].

Adolescents report that the major barriers to PA are environmental factors, which include insufficient time, opportunity, and access to resources [11–14]. Consequently, socioecological approaches that recognize the interplay of the individual and environment are best suited for eliciting PA behavioural change in adolescents [15–17]. Schools may be particularly apt environments for eliciting PA behavioural change among adolescents in Canada because adolescents attend school for substantial portions of their day and year; access to school programs is generally equitable, and adolescents from across cultures and socioeconomic strata are represented in schools; and schools have a formal role in the delivery of health and physical education (PE; [18, 19]). Previous reviews of school-based interventions for PA promotion for children and adolescents indicate the most promising approaches are multicomponent strategies targeting teachers' training, PE curricula development, community support, and policy modifications [11, 20, 21]. Despite this evidence, few studies examine the impact of large-scale multicomponent school-based PA or PE interventions targeting adolescents.

In response to the high prevalence of physical inactivity among adolescents in Canada, the province of Manitoba introduced a province-wide PE policy in September 2008 [22]. The new PE policy extended secondary school graduation requirements from 2 to 4 PE credits, mandating PE for grades 11 and 12 for the first time in Canada and impacting ~65,000 secondary school students in Manitoba annually [22]. The new grade 11 and 12 PE curriculum includes three components: (1) core, (2) flexible delivery, and (3) PA practicum. The core component is a minimum 25% (~30 hours) of the 110 credit hours spent in class studying health and personal planning, building competence for personal decisions around PA. The flexible delivery component allows schools and students to choose up to 25% (~25 hours) of the credit hours exploring selected areas of interest or specialization, either through an increase in the in-class time or through an increase in the out-of-class time, depending on local resources and needs. The PA practicum requires a minimum 50% (55 hours) of the 110 credit hours focused on participation in PA. The 55-hour time frame for the PA practicum was established based on the expectation that students need to accumulate a minimum of 30 minutes of moderate to vigorous PA (MVPA) per day on at least 5 days per week to achieve the course credit. Students can achieve the PA practicum through in-, out-, or a combination of in- and out-of-class time. The out-of-class physical activities eligible for credit include a wide variety of home, school, and community possibilities tailored to meet students' needs, interests, and opportunities and yet meet minimum standards of aerobic activity. The flexibility of the model is expected to minimize the time students spend away from academic studies and give students, families, schools, and communities more choices about how to include more PA in the lives of young people. This paper aims to examine the longitudinal changes in and the factors associated with the PA trajectories of adolescents in Manitoba during their tenure as secondary school students in the context of the described school-based PE policy.

## 2. Methods

### 2.1. Study Design

The data were collected as part of the Manitoba Increasing Physical Activity in Secondary Students (MIPASS) study. For the MIPASS study, students were recruited in a two-step process. Secondary schools (*n* = 31) that offered grades 9 to 12, had enrolment greater than 100, and were government operated were randomly selected in blocks to best represent the urban and rural geography of Manitoba [23]. In each selected school, a convenience sample of a grade 9 or 10 PE class was chosen for eligibility screening. All students in these PE classes were invited to participate in the study. Students who were absent from school for baseline recruitment were excluded from the study and data of students who were unable to perform regular activities of daily living or were living with chronic conditions that imposed limitations on PA were excluded from analyses. Written informed consent was obtained from parents and assent was obtained from students prior to study participation. Of the 596 grade 9 and 10 students recruited in April through June and October 2008, 533 (89.4%) students returned the accelerometers at baseline and were followed up once a year until 2011 or grade 12, whichever came first. Students who did not meet minimum accelerometer wear time requirement of 3 days and 8 hours at baseline (*n* = 65) or had an extreme MVPA baseline measurement (*n* = 21) were removed from analysis, leading to a final sample size of 447 (75.0%) students. Ethics approval was obtained from the Nursing and Education Research Ethics Board at the University of Manitoba.

## 3. Measures

### 3.1. Physical Activity

In the current study, PA was measured as minutes of MVPA per day using accelerometers and averaged over a 7-day period. At baseline, Actical (Respironics, Bend, OR) (*n* = 351) and ActiGraph accelerometers (CSA Actigraph, Pensacola, FL) (*n* = 96) were used; at follow-up collections, only the Actical accelerometers were used. Students were instructed to wear the accelerometer over their right hip on an elasticized belt for 7 consecutive days. They were to wear the device during their waking hours and take it off only for bathing and swimming. As described in a previous publication [24], raw PA counts were acquired in 15 seconds epochs and reintegrated into intensity categories using a specially designed software program (KineSoft, Saskatoon, Sask, Canada) that has been used in previous publications [25, 26]. Being consistent with previous research among adolescents, raw cut-points of ≥1500 counts per minute were used to classify MVPA, and the acceptable wear time criteria were defined as a minimum of 3 days and 480 registered minutes (i.e., 8 hours) of wear time at each data collection [27]. Eight hours of wear time was used in place of the more common threshold of 10 hours because sensitivity analyses of our data demonstrated that there were few differences between these criteria. Sequences of consecutive zero counts greater than or equal to 60 minutes were deemed nonwear and excluded from the analyses.

### 3.2. Definition of Time Periods

The baseline period (T0) was defined as April to October 2008. Although the baseline period slightly overlapped with the initial start of the policy (i.e., September 2008), it was assumed that the policy had not yet exerted detectable effects on student PA behaviour. Following the baseline period, time points were defined by the five-month school semesters typical in Manitoba occurring between February 2009 and December 2011, for example, September–January and February–June.

### 3.3. Student Demographics

Students' baseline demographics were collected using the Youth Health Survey assessing date of birth, grade, gender, and validated measures of self-reported height and weight that were used to calculate body mass index (BMI) [28]. Age was calculated as the difference between date of birth and date of first data collection. The International Obesity Task Force (IOTF) established age- and sex-specific cut-offs were used to classify students' BMI into healthy weight and overweight or obese [29]. Students who did not report height and/or weight were classified as missing BMI. BMI outside the range of 10–70 kg/m^2^ was deemed biologically implausible and was removed from analysis of BMI. Student baseline PA was ranked into tertiles, low, moderate, or high, based on their average minutes of MVPA per day captured from the accelerometer data collected at baseline.

### 3.4. School Neighbourhood Characteristics

School location and neighbourhood socioeconomic factors were identified to describe the level of resources available in the school neighbourhood for PA participation. These factors were identified by linking school postal codes to the 2006 Canadian census data and the Institut national de santé publique du Québec (INSPQ) material deprivation index at the dissemination area level, which corresponded to geographical areas of 400 to 700 people [23]. School locations were classified as urban or rural, with rural defined as having a 2006 census population of less than 10,000 [23]. School neighbourhoods were classified as having high or low socioeconomic status (SES), with low SES indicated by high levels of material deprivation. The validated material deprivation index incorporates 3 indicators from the 2006 Canadian census population aged 15 years and older: (1) the proportion without a high school diploma or equivalent, (2) employment to population ratio, and (3) average income [30]. The dissemination areas across the five Canadian regions were classified into quintiles, with those that were associated with school postal codes selected for analysis. Due to sample size, the quintiles were collapsed into high SES (quintiles 1–3) and low SES (quintiles 4-5) according to published methods [31]. This index was selected over single neighbourhood-level variables because the associations between single neighbourhood-level SES factors and adolescent PA in previous research were largely insignificant [32].

### 3.5. Statistical Analyses

Descriptive statistics of continuous and categorical variables were performed to describe the distribution of the variables at baseline across total study population, grade cohorts, and baseline PA. Individual growth curve modeling was employed to describe the variations in individual students' PA trajectories. This modeling technique allowed for independent adjustments for factors that were associated with students' baseline PA and those that affected their PA trajectories [33]. This technique can accommodate individuals who have unequal spacing in data collection and missing data in covariates and across time. To apply this technique, we first ran an unconditional linear growth curve model to identify the rate of PA change over the study period. An unconditional growth curve models the trajectories of PA over time, unadjusted for additional factors, which is used to evaluate the baseline amount of change. Unconditional growth curve models allow examination into the nature of the variation in PA (within or between people) and provide a baseline for evaluating the influence of additional predictor variables. Next, student and school neighbourhood characteristics were included in separate models to adjust for their effects on students' baseline PA and PA trajectories. The categories that were conceptualized to be protective against PA decline were selected as referent categories. Finally, multivariate linear growth curve models adjusting for characteristics of the students, school neighbourhood, and both student and school neighbourhood were run. Sensitivity analysis was also performed to examine for differences in PA across accelerometer models worn at baseline, students who did not provide follow-up measurements, and different quintile thresholds used to determine a cut-point for SES. Statistical significance was achieved at *P* < 0.05. Statistical analyses were performed using SAS version 9.2 (SAS Institute, Cary, NC).

## 4. Results

Table 1 describes the study cohort at baseline for the total study population, by grade cohort as well as by baseline PA. Of the 447 students with valid PA data, the average age at baseline was 15.2 ± 0.818 years, with 66.2% (*n* = 296) in grade 9. Slightly more than half of the study cohort were female (53.5%). The prevalence of students classified as overweight or obese was 13.6%, with 34.5% of the sample missing BMI due to lack of or erroneous reporting. Students with missing BMI were more likely to be older and male (*P* < 0.05). Further investigation did not detect differences in baseline PA across BMI categories (data not shown). Over two-thirds of the students attended schools in neighbourhoods of low SES, with approximately 30% of the students attending rural schools. Compared with students from urban schools, students at rural schools were more likely to demonstrate low baseline PA and to attend schools in neighbourhoods of low SES (*P* < 0.05).

### 4.1. Baseline Physical Activity

**Figure 1:**
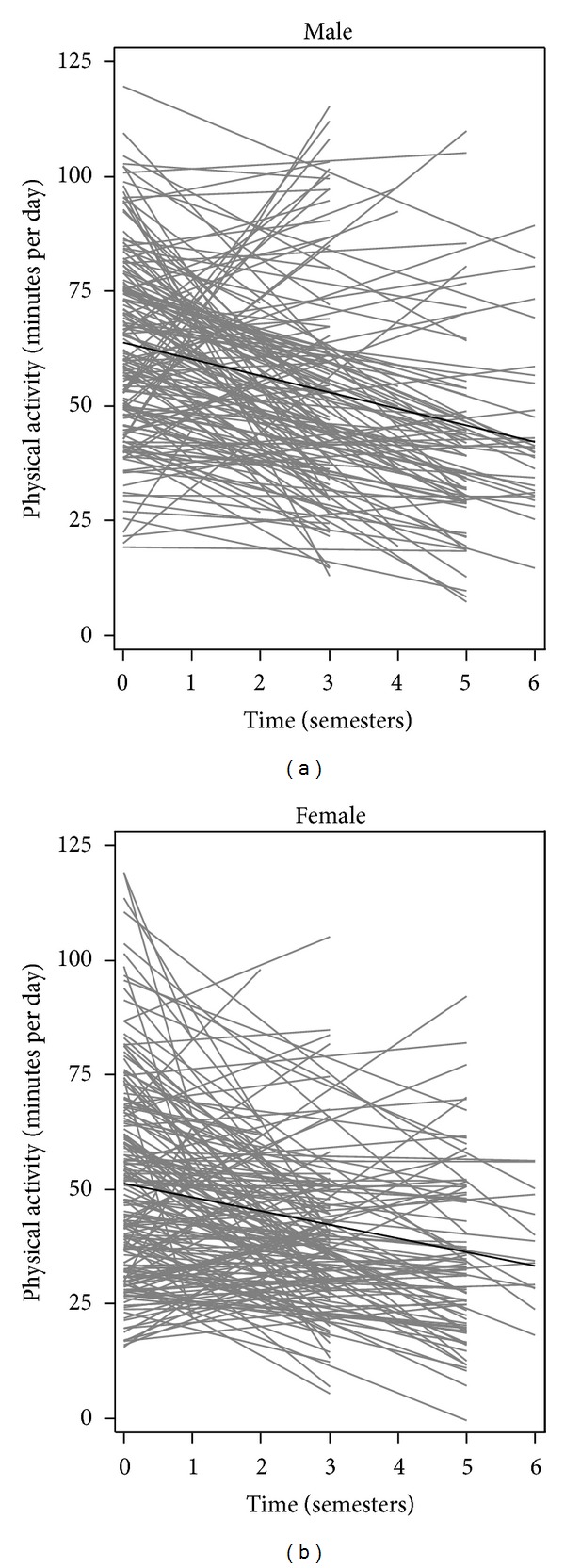
Physical activity trajectories of students from April 2008 to December 2011, by gender. Linear regressions of physical activity (minutes per day) across semesters for each student with baseline and at least 1 valid follow-up measurement, by gender, are shown in gray. Linear growth curves showing averaged baseline physical activity and averaged rate of change in physical activity among all students, by gender, are shown in black.

Objectively measured PA at baseline and follow-up periods is reported in Table 2. Each student contributed an average of 2.60 ± 1.11 assessments over the study duration. At baseline, the students accumulated an average of 58.3 ± 24.0 minutes of PA per day. Greater than 85% of the students accumulated the policy PA requirement of at least 30 minutes of MVPA per day at baseline while nearly 45% of the students accumulated the Canadian PA requirement of at least 60 minutes of MVPA per day at baseline [22, 34]. The baseline PA measures did not significantly differ across the grade cohorts. The growth curve model adjusted for gender only showed that gender PA levels differed significantly at baseline (Figure 1). Compared with males, females accumulated an average of 12.5 fewer minutes of PA per day (*P* < 0.0001). As shown in Table 3, students attending schools in rural areas accumulated an average of 10.6 fewer minutes of PA per day compared to students in urban schools (*P* < 0.0001). Similarly, students attending schools located in neighbourhoods of low SES accumulated an average of 9.1 fewer minutes of PA per day at baseline compared to students attending schools in neighbourhoods of high SES (*P* < 0.001). Overweight or obese students accumulated 1.9 minutes more of PA at baseline than students who had a healthy weight status, but the difference was not statistically significant. Results of the sensitivity analyses showed that the findings were similar across all student participants, including students who wore different accelerometer models at baseline and students who provided follow-up PA measurements compared to students who provided baseline PA measurements only. Using quintile cut-points of 1-2 for high and 3–5 for low material deprivation also did not alter study results.

### 4.2. Physical Activity Trajectories

Among the 340 students who provided baseline and at least one valid follow-up observation (76.1%), the last followup, on average, occurred 4.0 ± 1.4 semesters after baseline data collection, with 11.8% of last follow-up measurements occurring 1-2 semesters after baseline, 38.8% occurring 3-4 semesters after baseline, and 49.4% occurring 5-6 semesters after baseline. An average of 44.1 ± 23.7 minutes of PA was accumulated per day at the last followup. This represents a decrease of 13.4 ± 26.2 minutes of PA per day from baseline to last followup (Table 2). The proportion of students who accumulated at least 30 and 60 minutes of PA per day also fell to 67.9% and 20.9%, respectively. The average decrease in PA was not significantly different across the grade cohorts.

**Figure 2:**
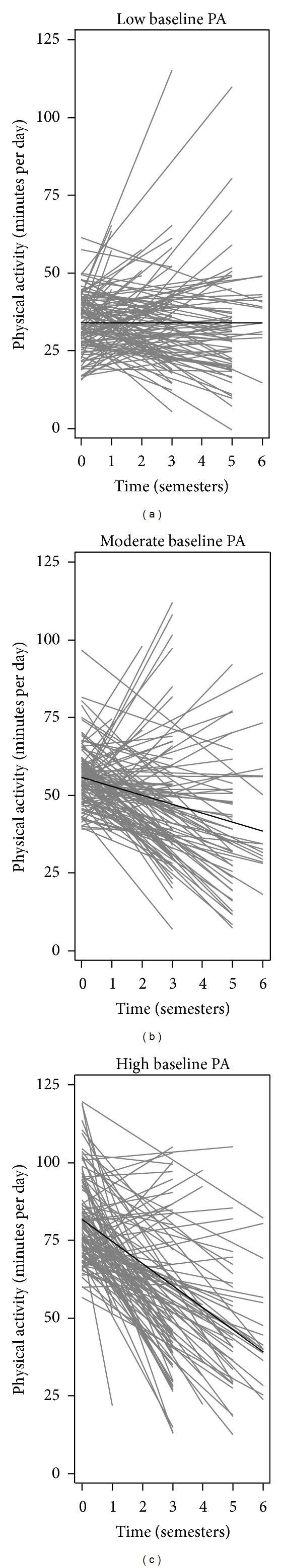
Physical activity trajectories of students from April 2008 to December 2011, by baseline physical activity. Linear regressions of physical activity (minutes per day) across semesters for each student with baseline and at least 1 valid follow-up measurement, by baseline PA, are shown in gray. Linear growth curves showing averaged baseline physical activity and averaged rate of change in physical activity among all students, by baseline PA, are shown in black.

After adjusting for time of data collection (i.e., T0–T6) in the unconditional linear growth model, the average rate of decline in PA among students was 3.2 minutes per semester (*P* < 0.0001) (data not shown). This corresponds to an average PA decline of 11.3% over a school year. As shown in Figure 2, further adjustment for baseline PA showed that students with low or moderate baseline PA had slower average rates of PA decline than students with high baseline PA (*P* < 0.0001). In a separate model adjusting for time and school location, compared with students attending urban schools, those attending rural schools showed slower rate of decline in PA (*P* = 0.02). Similarly, the model adjusting for time and SES indicated that students attending schools in areas of low SES also showed a slower rate of decline in PA than students attending schools in areas of high SES (*P* = 0.0086). The students' PA trajectories over time did not significantly differ by gender (*P* = 0.32, Figure 2) or across BMI status (*P* = 0.39) when examined in separate models.

In the multivariate linear growth curve model (Table 4), we adjusted for gender, baseline PA, BMI status, school location, and neighbourhood SES on students' baseline PA and PA trajectories. Among the student and school neighbourhood factors, the association of students' baseline PA with their PA trajectories was most statistically significant. Compared with students who had high baseline PA, the adjusted PA trajectories of students with low and moderate baseline PA declined less steeply, differing by an average of 7.1 minutes per semester (*P* < 0.0001) and 4.2 minutes per semester (*P* < 0.0001), respectively. Students attending schools located in neighbourhoods of low SES also had a slower adjusted rate of decline in PA than those attending schools in areas of high SES, with the rates of the two groups differing by an average of 1.5 minutes per semester (*P* = 0.0432). The difference in the adjusted PA trajectories across gender, BMI status, and school location was not statistically significant.

## 5. Discussion

To our knowledge, this is the first study to examine the PA trajectories of adolescents in Canada in the context of a province-wide PE policy aimed at increasing PA. The findings indicate that, despite the PE policy in Manitoba, the PA trajectories of adolescents declined over time as the adolescents advanced to higher grade levels; however, the decline in PA was attenuated among adolescents with low and moderate baseline PA and who attended schools in neighbourhoods of low SES.

In the current study, adolescents' PA trajectories across the secondary school tenure are strongly influenced by their baseline PA. The decline in adolescent PA over time was attenuated among participants with low and moderate baseline PA compared to students with high baseline PA. Previous longitudinal MVPA data among youth have shown baseline PA to be the strongest predictor of future PA patterns, whereby students with relatively low PA at baseline in early adolescence continue to participate in lower levels of PA in later adolescence [35]. Based on these previous results, experts recommend PA interventions targeting children to establish habitual PA behaviour that can carry over into adolescence [35]. Findings of the current study suggest, however, that the rate of decline in adolescents' PA trajectories over the course of secondary school varies depending on baseline PA, with steeper declines among adolescents with high baseline PA and slower declines among adolescents with low and moderate baseline PA. In other words, higher PA levels in early adolescence were not preventive of declines in PA in later adolescence. Instead, PA behaviour appears to stabilize towards the end of secondary school to approximately 30 minutes of MVPA per day, regardless of PA level near the start of secondary school. This is somewhat encouraging as 30 minutes of MVPA per day produces some health benefits among adolescents and is equivalent to the Canadian PA guidelines for adults aged 18–64 years [4, 36]. It is uncertain, however, if the amount of PA achieved by adolescents in the current study at the end of secondary school is associated with the PE policy requirement of a minimum of 30 minutes of MVPA per day and the tendency for students to conform to school policy expectations. Another possible explanation for adolescents' PA behaviour to stabilize over time is measurement error. The observed trend in adolescent PA may be heavily influenced by regression to the mean level of PA. For instance, a single 7-day baseline PA measure was recorded, and adolescents with high PA at baseline may have been measured over a week where they were particularly active. Therefore, at follow-up measurements, they were observed to be less active. The opposite could also be true for adolescents with low PA measures at baseline. A double baseline measure may have helped researchers to better understand the students' habitual PA behaviour in grades 9 and 10 and reduced potential biases due to social desirability. Nevertheless, interventions to support adolescent PA regardless of lifestyle habits of PA prior to or in early adolescence, in addition to child-focused PA interventions, appear necessary to achieve and maintain healthy levels of activity.

The decline in adolescent PA over the course of secondary school was significantly less steep among students attending schools located in neighbourhoods of low SES compared to high SES. This finding contradicts the majority of existing evidence suggesting adolescents residing in neighbourhoods of lower SES engage in less PA than those in neighbourhoods of higher SES [37–39]. One explanation provided for the discrepancy in adolescent PA across neighbourhood SES is that adolescents living in neighbourhoods of lower SES have limited access to opportunities and resources for PA [40]. Therefore, it is possible that the slower rate of decline in PA among adolescents attending schools in neighbourhoods of low SES in the current study is because the new PE policy in Manitoba increased access to and availability of PA opportunities and resources for these adolescents. Indeed, findings from interviews conducted with teachers in Manitoba 1-year following policy implementation as part of the larger MIPASS study indicate that, as a result of the PE policy, school, and community stakeholders recognized a gap in PA programming targeting older adolescents. To fill this gap, new PA opportunities and resources for older adolescents in schools and in surrounding community organizations in Manitoba were developed. It is not possible from the current study, however, to determine if students engaged in the new school and community-based PA programming to fulfill the PA practicum requirement of the PE policy. Future research should consider inviting students to complete PA diaries and employing accelerometers with built-in GPS units in order to better understand where and how students are accumulating or participating in PA.

A number of limitations in the current study should be noted. Although participating schools were randomly selected across Manitoba, the Grades 9 and 10 students who participanted were conveniently sampled within PE classes of these schools and may in part contribute to the relatively large proportion of participants in the current study who had a healthy weight and who met the Canadian PA recommendation of at least 60 minutes of MVPA per day at both baseline and last follow-up measures compared to the nationally representative sample in the Canadian Health Measures Survey [5, 34]. This sampling strategy may limit the generalizability of the findings beyond this cohort; however, PE is mandatory in Manitoba for grade 9 and 10 students and therefore student recruitment should not bias towards subpopulations who favour PA. Additionally, a control or comparison group not exposed to the province-wide PE policy was not included in this study. Including a control or comparison group in the current study would allow for inferences to be made about the impact of the PE policy in Manitoba on adolescent PA. Yet, given that adolescent PA can be highly dependent on environmental factors the extent to which PA trajectories of adolescents outside of Manitoba in the Canadian context can be appropriately compared to the study population may be limited [11, 32, 38]. Nevertheless, this study was able to capture students' PA prepolicy and at multiple time points postpolicy which enabled examining factors related to adolescents' PA trajectories using growth curve modeling. Moreover, this study included an objective measurement of PA across a relatively large sample with an even distribution of males and females during an important developmental period in the life course.

## 6. Conclusion 

School-based PE policies targeting adolescent PA may support adolescents in maintaining healthy levels of PA throughout adolescence, regardless of PA patterns established during childhood or early adolescence. Moreover, such PE policies may address gaps in PA opportunities and programming for adolescents, especially among those attending schools in lower SES neighbourhoods with potentially fewer PA resources. Further prospective and intervention research examining the impact of school-based PE policies specifically targeting adolescent PA seems warranted if we are to positively influence the PA trajectories of young people. Moreover, research using GPS units and PA diaries in addition to objective measures of PA may add value to better understand where and how adolescents are accumulating or participating in PA over the course of their secondary school tenure.

## Figures and Tables

**Table 1:** Baseline characteristics across grade cohorts, total study sample, and baseline physical activity.

Baseline student characteristics	Grade 9 (*n* = 296)		Grade 10 (*n* = 151)		Total (*n* = 447)		Baseline physical activity (*n* = 447)	
	*n*	%	*n*	%	*n*	%	mean	SD
Age (years), mean ± SD	14.8	±0.705	15.9	±0.419	15.2	±0.818	N.A.	
Gender								
Male	142	(48.0)	62	(41.1)	204	(45.6)	64.6	±23.3
Female	151	(51.0)	88	(58.3)	239	(53.5)	53.0	±23.0

BMI category								
Overweight or obese	40	(13.5)	21	(13.9)	61	(13.6)	58.2	±23.8
Healthy weight	143	(48.3)	85	(56.3)	228	(51.0)	58.0	±23.5
Missing	109	(36.8)	45	(29.8)	154	(34.5)	58.6	±24.9

Baseline physical activity level								
Low	103	(34.8)	46	(30.5)	149	(33.3)	32.8	±8.4
Moderate	89	(30.1)	60	(39.7)	149	(33.3)	56.4	±6.4
High	104	(35.1)	45	(29.8)	149	(33.3)	85.8	±14.2

Baseline school neighbourhood characteristics								

School location								
Rural	73	(24.7)	60	(39.7)	133	(29.8)	48.8	±19.6
Urban	223	(75.3)	91	(60.3)	314	(70.2)	62.4	±24.5

Socioeconomic status								
Low	204	(68.9)	104	(68.9)	308	(68.9)	55.3	±22.9
High	92	(31.1)	47	(31.1)	139	(31.1)	65.0	±24.9

Proportions may not add up to 100% due to missing values.

N.A.: not applicable.

**Table 2:** Objectively measured physical activity at baseline and follow-up periods across grade cohorts and total study sample.

	Grade 9 (*n* = 296)		Grade 10 (*n* = 151)		Total (*n* = 447)		*P* value
	*n*	%	*n*	%	*n*	%	
Students with valid PA at each time point							
T0: Prepolicy	296	(100.0)	151	(100.0)	477	(100.0)	N.A.
T1: February 2009–June 2009	99	(33.4)	68	(45.0)	167	(37.4)	N.A.
T2: September 2009–January 2010	73	(24.7)	21	(13.9)	94	(21.0)	N.A.
T3: February 2010–June 2010	138	(46.6)	94	(62.3)	232	(51.9)	N.A.
T4: September 2010–January 2011	54	(18.2)	0	(0.0)	54	(12.1)	N.A.
T5: February 2011–June 2011	122	(41.2)	2	(0.0)	124	(27.7)	N.A.
T6: September 2011–December 2011	44	(14.9)	0	(0.0)	44	(0.09)	N.A.

Time points contributed per student, mean ± SD	2.79	±1.18	2.23	±0.850	2.60	±1.11	<0.0001^a^

Baseline PA							
Students with baseline data	296	(100.0)	151	(100.0)	447	(100.0)	N.A.
Minutes per day, mean ± SD	58.7	±24.1	57.6	±23.8	58.3	±24.0	0.5890^a^
Accumulated ≥ 30 min/day	265	(89.5)	131	(86.8)	396	(88.6)	0.3833^b^
Accumulated ≥ 60 min/day	138	(46.6)	61	(40.4)	199	(44.5)	0.2104^b^

PA at students' last followup							
Students with ≥ 1 followup	230	(77.7)	110	(72.9)	340	(76.1)	0.3304^b^
Minutes per day, mean ± SD	42.6	±23.5	47.2	±23.9	44.1	±23.7	0.0485^a^
Accumulated ≥ 30 min/day (% with ≥ 1 followup)	149	(64.8)	82	(74.6)	231	(67.9)	0.0712^b^
Accumulated ≥ 60 min/day (% with ≥ 1 followup)	46	(20.0)	25	(22.7)	71	(20.9)	0.5627^b^

PA change from baseline to last followup							
Students with ≥ 1 followup	230	(77.7)	110	(72.9)	340	(76.1)	0.3304^b^
Change (min/day), mean ± SD	−14.6	±24.7	−11.1	±29.0	−13.4	±26.2	0.2701^a^
Percentage change (%), mean ± SD	−18.7	±48.8	−10.0	±51.0	−15.9	±49.6	0.1871^a^

^a^Wilcoxon two-sample test used to compare across grade cohorts.

^b^Chi-square test used to compare across grade cohorts.

N.A.: denotes not applicable.

**Table 3:** Unadjusted linear growth curve modeling examining estimated effect on students' baseline physical activity and physical activity trajectories.

	Estimated effect on baseline physical activity (minutes per day)		Estimated effect on physical activity trajectories (minutes per day per semester)	
	Fixed effect	*P* value	Fixed Effect	*P* value
Student Characteristics				

Gender				
Female	−12.5 (2.04)	<0.0001	0.596 (0.598)	0.3194
Male	Referent		Referent	

Baseline physical activity				
Low	−47.7 (1.50)	<0.0001	7.11 (0.707)	<0.0001
Moderate	−25.8 (1.51)	<0.0001	4.20 (0.719)	<0.0001
High	Referent		Referent	

BMI status				
Overweight or obese	1.92 (3.21)	0.5512	−0.785 (0.901)	0.3894
Healthy weight	Referent		Referent	
Missing	1.88 (2.35)	0.4252	−0.0527 (0.657)	0.9362

School neighbourhood characteristics				

School location				
Rural	−10.6 (2.25)	<0.0001	1.49 (0.658)	0.0234
Urban	Referent		Referent	

Socioeconomic status				
Low	−9.09 (2.27)	<0.0001	1.75 (0.664)	0.0086
High	Referent		Referent	

Fixed effects of intercepts for each model are not shown.

Standard errors are shown in parentheses.

Referent categories are identified as “referent.”

**Table 4:** Multivariate growth curve modeling examining estimated effect on students' baseline physical activity and physical activity trajectories when adjusted for student and school neighbourhood characteristics.

	Model 1		Model 2		Model 3	
	Student characteristics		School neighbourhood characteristics		Student and school neighbourhood characteristics	
	Fixed effect	*P* value	Fixed effect	*P* value	Fixed effect	*P* value
Estimated effect on baseline physical activity (minutes per day)						

Gender						
Female	−3.81 (1.27)	0.0029			−4.20 (1.28)	0.0011
Male	Referent				Referent	

Baseline physical activity						
Low	−46.5 (1.54)	<0.0001			−46.0 (1.59)	<0.0001
Moderate	−25.1 (1.51)	<0.0001			−25.0 (1.53)	<0.0001
High	Referent				Referent	

BMI status						
Overweight or obese	2.32 (1.81)	0.1999			2.56 (1.80)	0.1562
Healthy weight	Referent				Referent	
Missing	1.48 (1.38)	0.2815			1.41 (1.37)	0.3036

School location						
Rural			−8.25 (2.49)	0.0010	0.680 (1.48)	0.6465
Urban			Referent		Referent	

Socioeconomic status						
Low			−5.46 (2.50)	0.0295	−3.47 (1.50)	0.0216
High			Referent		Referent	

Estimated effect on physical activity trajectories (minutes per day per semester)						

Gender						
Female	−0.885 (0.608)	0.1463			−0.735 (0.612)	0.2304
Male	Referent				Referent	

Baseline physical activity						
Low	7.34 (0.741)	<0.0001			7.08 (0.759)	<0.0001
Moderate	4.24 (0.735)	<0.0001			4.19 (0.749)	<0.0001
High	Referent				Referent	

BMI status						
Overweight or obese	−1.03 (0.866)	0.2343			−1.19 (0.866)	0.1711
Healthy weight	Referent				Referent	
Missing	−0.00292 (0.653)	0.9964			0.0645 (0.652)	0.9213

School location						
Rural			0.942 (0.716)	0.1888	−0.0316 (0.705)	0.9643
Urban			Referent		Referent	

Socioeconomic status						
Low			1.35 (0.724)	0.0629	1.46 (0.722)	0.0432
High			Referent		Referent	

Fixed effects of intercepts for each model are not shown.

Standard errors are shown in parentheses.

Referent categories are identified as “referent.”

## References

[1] Roberts K, Shields M, de Groh M, Aziz A, Gilbert J (2012). Overweight and obesity in children and adolescents: results from the 2009 to 2011 Canadian Health Measures Survey. *Health Reports*.

[2] Ball GDC, McCargar LJ (2003). Childhood obesity in Canada: a review of prevalence estimates and risk factors for cardiovascular diseases and type 2 diabetes. *Canadian Journal of Applied Physiology*.

[3] Franks PW, Hanson RL, Knowler WC, Sievers ML, Bennett PH, Looker HC (2010). Childhood obesity, other cardiovascular risk factors, and premature death. *The New England Journal of Medicine*.

[4] Janssen I, LeBlanc AG (2010). Systematic review of the health benefits of physical activity and fitness in school-aged children and youth. *International Journal of Behavioral Nutrition and Physical Activity*.

[5] Colley RC, Garriguet D, Janssen I, Craig CL, Clarke J, Tremblay MS (2011). Physical activity of canadian children and youth: accelerometer results from the 2007 to 2009 canadian health measures survey. *Health Reports*.

[6] Kimm SYS, Glynn NW, Kriska AM (2002). Decline in physical activity in black girls and white girls during adolescence. *The New England Journal of Medicine*.

[7] Kimm SYS, Glynn NW, Obarzanek E (2005). Relation between the changes in physical activity and body-mass index during adolescence: a Multicentre Longitudinal Study. *The Lancet*.

[8] Shields M (2006). Overweight and obesity among children and youth. *Health Reports*.

[9] Herman KM, Craig CL, Gauvin L, Katzmarzyk PT (2009). Tracking of obesity and physical activity from childhood to adulthood: the Physical Activity Longitudinal Study. *International Journal of Pediatric Obesity*.

[10] Katzmarzyk PT, Janssen I (2004). The economic costs associated with physical inactivity and obesity in canada: an update. *Canadian Journal of Applied Physiology*.

[11] Sallis JF, Bauman A, Pratt M (1998). Environmental and policy interventions to promote physical activity. *American Journal of Preventive Medicine*.

[12] McKenzie TL, Marshall SJ, Sallis JF, Conway TL (2000). Leisure-time physical activity in school environments: an observational study using SOPLAY. *Preventive Medicine*.

[13] Hill JO, Peters JC (1998). Environmental contributions to the obesity epidemic. *Science*.

[14] French SA, Story M, Jeffery RW (2001). Environmental influences on eating and physical activity. *Annual Review of Public Health*.

[15] McLeroy KR, Bibeau D, Steckler A, Glanz K (1988). An ecological perspective on health promotion programs. *Health Education Quarterly*.

[16] Sallis J, Owen N, Glanz K, Lewis F, Latimer AE (1997). Ecological models. *Health Behaviour and Health Education: Theory, Research and Practice*.

[17] Pellmar TC, Brandt EN, Baird MA (2002). Health and behavior: the interplay of biological, behavioral, and social influences: summary of an Institute of Medicine report. *American Journal of Health Promotion*.

[18] Fox KR, Cooper A, McKenna J (2004). The school and promotion of children’s health-enhancing physical activity: perspectives from the United Kingdom. *Journal of Teaching in Physical Education*.

[19] Naylor P-J, Scott J, Drummond J, Bridgewater L, McKay HA, Panagiotopoulos C (2010). Implementing a whole school physical activity and healthy eating model in rural and remote first nations schools: a process evaluation of action schools! BC. *Rural and Remote Health*.

[20] Sallis JF, Conway TL, Prochaska JJ, McKenzie TL, Marshall SJ, Brown M (2001). The association of school environments with youth physical activity. *American Journal of Public Health*.

[21] Felton G, Saunders RP, Ward DS, Dishman RK, Dowda M, Pate RR (2005). Promoting physical activity in girls: a case study of one school’s success. *Journal of School Health*.

[22] http://www.edu.gov.mb.ca/k12/docs/policy/imp_pehe/document.pdf.

[23] http://www12.statcan.gc.ca/census-recensement/2006/dp-pd/hlt/97-550/Index.cfm?TPL=P1C&Page=RETR&LANG=Eng&T=601&PR=46&S=0&O=D&RPP=25.

[24] Comte M, Hobin E, Majumdar SR, Plotnikoff RC, Ball GD, McGavock J (2013). Patterns of weekday and weekend physical activity in youth in 2 Canadian provinces. *Applied Physiology, Nutrition, and Metabolism*.

[25] Esliger DW, Tremblay MS (2007). Physical activity and inactivity profiling: the next generation. *Canadian Journal of Public Health*.

[26] Esliger DW, Tremblay MS, Copeland JL, Barnes JD, Huntington GE, Bassett DR (2010). Physical activity profile of old order amish, mennonite, and contemporary children. *Medicine and Science in Sports and Exercise*.

[27] Hay J, Maximova K, Durksen A, Carson V, Rinaldi RL, Torrance B (2012). Physical activity intensity and cardiometabolic risk in youth. *Archives of Pediatrics and Adolescent Medicine*.

[28] Wong SL, Leatherdale ST, Manske S (2006). Reliability and validity of a school-based physical activity questionnaire. *Medicine and Science in Sports and Exercise*.

[29] Cole TJ, Bellizzi MC, Flegal KM, Dietz WH (2000). Establishing a standard definition for child overweight and obesity worldwide: international survey. *British Medical Journal*.

[30] Gamache P, Pampalon R, Hamel D (2010). *Methodological Guide-the Material and Social Deprivation Index: A Summary*.

[31] Carter MA, Dubois L, Tremblay MS, Taljaard M, Jones BL (2012). Trajectories of childhood weight gain: the relative importance of local environment versus individual social and early life factors. *PloS ONE*.

[32] Stalsberg R, Pedersen AV (2010). Effects of socioeconomic status on the physical activity in adolescents: a systematic review of the evidence. *Scandinavian Journal of Medicine and Science in Sports*.

[33] Singer JD, Willett JB (2003). *Applied Longitudinal Data Analysis: Modeling Change and Event Occurrence: Modeling Change and Event Occurrence*.

[34] Canadian Society for Exercise Physiology. Canadian physical activity guidelines for youth ages 12–17 years. http://www.csep.ca/CMFiles/Guidelines/CSEP_PAGuidelines_youth_en.pdf.

[35] Hearst MO, Patnode CD, Sirard JR, Farbakhsh K, Lytle LA (2012). Multilevel predictors of adolescent physical activity: a longitudinal analysis. *International Journal of Behavioral Nutrition and Physical Activity*.

[36] Canadian Society for Exercise Physiology. Canadian physical activity guidelines for adults ages 18–64 years. http://www.csep.ca/CMFiles/Guidelines/CSEP_PAGuidelines_youth_en.pdf.

[37] Hanson MD, Chen E (2007). Socioeconomic status and health behaviors in adolescence: a review of the literature. *Journal of Behavioral Medicine*.

[38] Ferreira I, Van Der Horst K, Wendel-Vos W, Kremers S, Van Lenthe FJ, Brug J (2007). Environmental correlates of physical activity in youth—a review and update. *Obesity Reviews*.

[39] Salonna F, Van Dijk JP, Geckova AM, Sleskova M, Groothoff JW, Reijneveld SA (2008). Social inequalities in changes in health-related behaviour among Slovak adolescents aged between 15 and 19: a longitudinal study. *BMC Public Health*.

[40] Gordon-Larsen P, Nelson MC, Page P, Popkin BM (2006). Inequality in the built environment underlies key health disparities in physical activity and obesity. *Pediatrics*.

